# Supplementary material for: Niche Separation Increases With Genetic Distance Among Bloom-Forming Cyanobacteria
Source: Front Microbiol. 2018 Mar 27;9:438. doi: 10.3389/fmicb.2018.00438 (PMC5880894; doi:10.3389/fmicb.2018.00438)
Supplement: Supplementary file 1 [file Data_Sheet_1.pdf]

Niche separation increases with genetic distance among bloom-forming cyanobacteria

Authors

Nicolas Tromas[1*#], Zofia E. Taranu[2*], Bryan D Martin[3], Amy Willis[4], Nathalie Fortin[5], Charles W. Greer[5] and B. Jesse Shapiro[1#]

SUPPLEMENTARY FIGURES AND TABLES

Table S1. Significant relationships between MED nodes and environmental variables

Predictor	Node #	R2-adj	p-value	Degree	Genera
**PP**	D2282	0.13	0.0008	linear	Dolicho
	D5630	0.12	0.0013	linear	Dolicho
	M5733	0.05	0.0558	non-linear	Micro
	M5736	0.06	0.0200	linear	Micro
**PN**	D2282	0.05	0.0322	linear	Dolicho
	D5630	0.09	0.0046	linear	Dolicho
	M5733	0.05	0.0303	linear	Micro
	M5736	0.06	0.0167	linear	Micro
**DP**	M5732	0.04	0.0429	linear	Micro
	M5734	0.04	0.0461	linear	Micro
	M5738	0.05	0.0280	linear	Micro
**DN**	D2424	0.16	0.0002	linear	Dolicho
	D5630	0.05	0.0342	linear	Dolicho
	M5732	0.06	0.0248	linear	Micro
	M5734	0.06	0.0184	linear	Micro
**Precip**	D2282	0.10	0.0029	linear	Dolicho
	D5630	0.10	0.0034	linear	Dolicho
**Temp**	M5734	0.07	0.0124	linear	Micro
	M5738	0.09	0.0065	linear	Micro
**MC**	M5734	0.07	0.0271	non-linear	Micro

Table S2. Significant relationships between genera and environmental variables

Predictor	Node #	R2-adj	p-value	Degree
**PP**	Dolicho	0.15	0.0005	linear
**PN**	Dolicho	0.16	0.0009	non-linear
**DP**	Micro	0.06	0.0137	linear
**DN**	Dolicho	0.07	0.0241	linear

Table S3. Significant (bold font) and non-significant (grey font) correlations between fitted values from LVMs corresponding to Figures S6/S7.

Predictor	Node pair	r	gen dist
**PP**	**2282-5630**	**-0.91**	**0.047**
	2424-5630	-0.57	0.012
	5505-2282	-0.30	0.02
	5631-2282	-0.21	0.043
	2424-5505	0.21	0.016
	2424-5631	0.23	0.008
	5505-5630	0.65	0.028
	**2424-2282**	**0.84**	**0.036**
	5631-5505	0.80	0.024
	5631-5630	0.60	0.004
**PN**	**2282-5630**	**-0.90**	**0.047**
	2424-5505	-0.19	0.016
	2424-5630	-0.49	0.012
	5505-2282	-0.70	0.02
	5631-2282	-0.40	0.043
	2424-5631	0.07	0.008
	5505-5630	0.88	0.028
	2424-2282	0.71	0.036
	5631-5505	0.73	0.024
	5631-5630	0.70	0.004
**DP**	2282-5630	-0.68	0.047
	2424-5630	-0.06	0.012
	5505-5630	-0.17	0.028
	2424-2282	-0.13	0.036
	5505-2282	0.00	0.02
	5631-5630	-0.33	0.004
	2424-5505	0.68	0.016

	2424-5631	0.64	0.008
	5631-2282	0.17	0.043
	5631-5505	0.47	0.024
**DN**	2282-5630	-0.43	0.047
	2424-2282	-0.37	0.036
	5505-2282	-0.62	0.02
	**2424-5505**	**0.83**	**0.016**
	**2424-5630**	**0.93**	**0.012**
	2424-5631	0.70	0.008
	**5505-5630**	**0.85**	**0.028**
	5631-2282	0.19	0.043
	5631-5505	0.19	0.024
	5631-5630	0.57	0.004
**Precip**	**2282-5630**	**-0.85**	**0.047**
	2424-5631	-0.62	0.008
	2424-2282	-0.44	0.036
	5505-2282	-0.87	0.02
	5631-2282	-0.20	0.043
	5631-5630	-0.20	0.004
	2424-5505	0.38	0.016
	**2424-5630**	**0.81**	**0.012**
	5505-5630	0.77	0.028
	5631-5505	0.19	0.024
**Temp**	2282-5630	-0.33	0.047
	2424-5631	-0.04	0.008
	2424-2282	-0.34	0.036
	5505-2282	-0.15	0.02
	5631-5505	-0.09	0.024
	5631-5630	-0.12	0.004
	2424-5505	0.56	0.016
	2424-5630	0.74	0.012
	5505-5630	0.41	0.028
	5631-2282	0.02	0.043
**MC**	2282-5630	0.00	0.047
	2424-5630	-0.01	0.012
	5505-5630	-0.16	0.028
	2424-2282	-0.35	0.036
	5505-2282	-0.23	0.02
	5631-2282	-0.11	0.043
	5631-5630	-0.50	0.004
	2424-5505	0.79	0.016
	2424-5631	0.45	0.008
	5631-5505	0.51	0.024

Table S4. Significant ($p < 0.10$) relationships between LVM co-responses and genetic distance as shown in Figures 4 and S8.

Predictor	R2-adj	p-value	Degree	Genera
**PP**	0.69	0.0184	cubic	Dolicho
**PN**	0.63	0.0280	cubic	Dolicho
**DN**	0.25	0.0791	linear	Dolicho
**MC**	0.29	0.0231	linear	Micro

Table S5. Table of the taxa present in both *D* and *M* networks (Figure S9). In bold are represented taxa that are co-occurrent with both *D* and *M*.

Nodes	Taxonomy
2966	p_Bacteroidetes| c_[Saprospirae]| o_[Saprospirales]| bacI| bacI-A| unclassified
3667	p_Bacteroidetes| c_Cytophagia| o_Cytophagales| f_Cytophagaceae| unclassified| unclassified
5983	p_Bacteroidetes| c_Cytophagia| o_Cytophagales| f_Cytophagaceae| unclassified| unclassified
5984	p_Bacteroidetes| c_Cytophagia| o_Cytophagales| f_Cytophagaceae| unclassified| unclassified
6317	p_Bacteroidetes| c_Flavobacteriia| o_Flavobacteriales| bacII| bacII-A| unclassified
6331	p_Bacteroidetes| c_Sphingobacteriia| o_Sphingobacteriales| unclassified| unclassified| unclassified
4062	p_Chloroflexi| c_SL56| unclassified| unclassified| unclassified| unclassified
1059	p_Cyanobacteria| c_Synechococcophycideae| o_Pseudanabaenales| f_Pseudanabaenaceae| g_Pseudanabaena| unclassified
**1061**	**p_Cyanobacteria| c_Synechococcophycideae| o_Pseudanabaenales| f_Pseudanabaenaceae| g_Pseudanabaena| unclassified**
2392	p_Gemmatimonadetes| c_Gemmatimonadetes| o_Gemmatimonadales| unclassified| unclassified| unclassified
**4674**	**p_Proteobacteria| c_Alphaproteobacteria| o_Caulobacterales| f_Caulobacteraceae| g_Phenylobacterium| unclassified**
4737	p_Proteobacteria| c_Alphaproteobacteria| o_Rhizobiales| alfI| alfI-A| alfI-A1
3705	p_Proteobacteria| c_Alphaproteobacteria| o_Rhizobiales| f_Xanthobacteraceae| unclassified| unclassified
3726	p_Proteobacteria| c_Alphaproteobacteria| o_Rhizobiales| unclassified| unclassified| unclassified
5294	p_Proteobacteria| c_Alphaproteobacteria| o_Rhodospirillales| alfVIII| unclassified| unclassified

5471	p_Proteobacteria| c_Alphaproteobacteria| o_Rhodospirillales| alfVIII| unclassified| unclassified
**7272**	**p_Proteobacteria| c_Alphaproteobacteria| o_Rhodospirillales| alfVIII| unclassified| unclassified**
7273	p_Proteobacteria| c_Alphaproteobacteria| o_Rhodospirillales| alfVIII| unclassified| unclassified
7655	p_Proteobacteria| c_Betaproteobacteria| o_Burkholderiales| betVII| unclassified| unclassified
5221	p_Proteobacteria| c_Betaproteobacteria| o_Burkholderiales| f_Comamonadaceae| g_Rubrivivax| unclassified
4374	p_Proteobacteria| c_Betaproteobacteria| o_Burkholderiales| f_Comamonadaceae| unclassified| unclassified
**4756**	**p_Proteobacteria| c_Betaproteobacteria| o_undefined| betV| betV-A| betV-A1**
4757	p_Proteobacteria| c_Betaproteobacteria| o_undefined| betV| betV-A| betV-A1
4377	p_Proteobacteria| c_Betaproteobacteria| unclassified| unclassified| unclassified| unclassified
6996	p_Proteobacteria| c_Betaproteobacteria| unclassified| unclassified| unclassified| unclassified
1286	p_Verrucomicrobia| c_[Spartobacteria]| o_[Chthoniobacterales]| verI-A| Xip-A1| unclassified

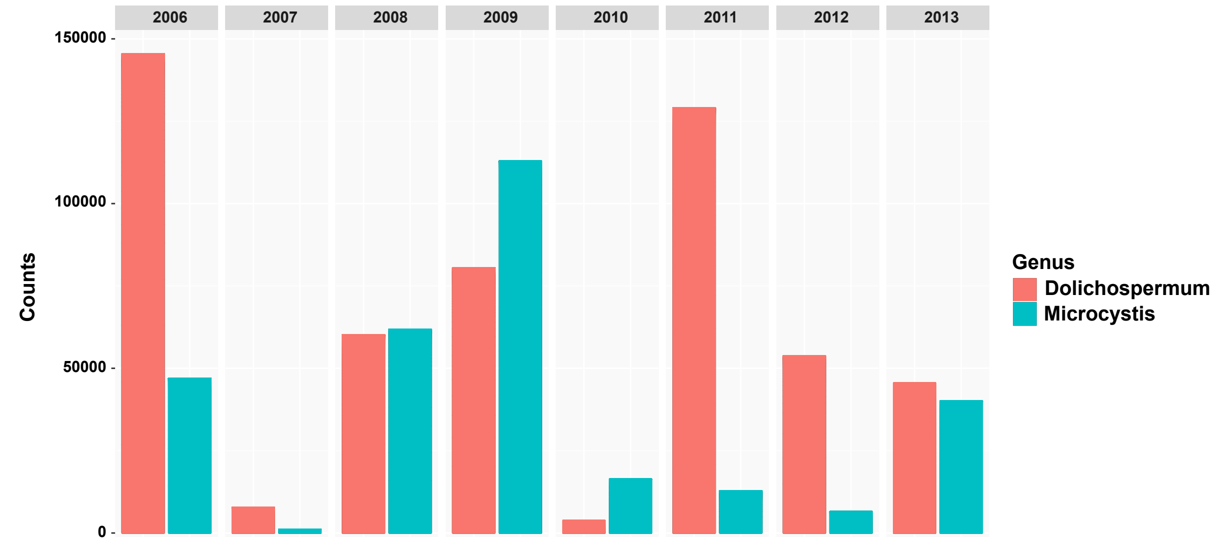

Figure S1. Barplot of the absolute abundance (counts of sequence reads) of *Dolichospermum* and *Microcystis* genera in Lake Champlain's Bay Missisquoi from 2006 to 2013.

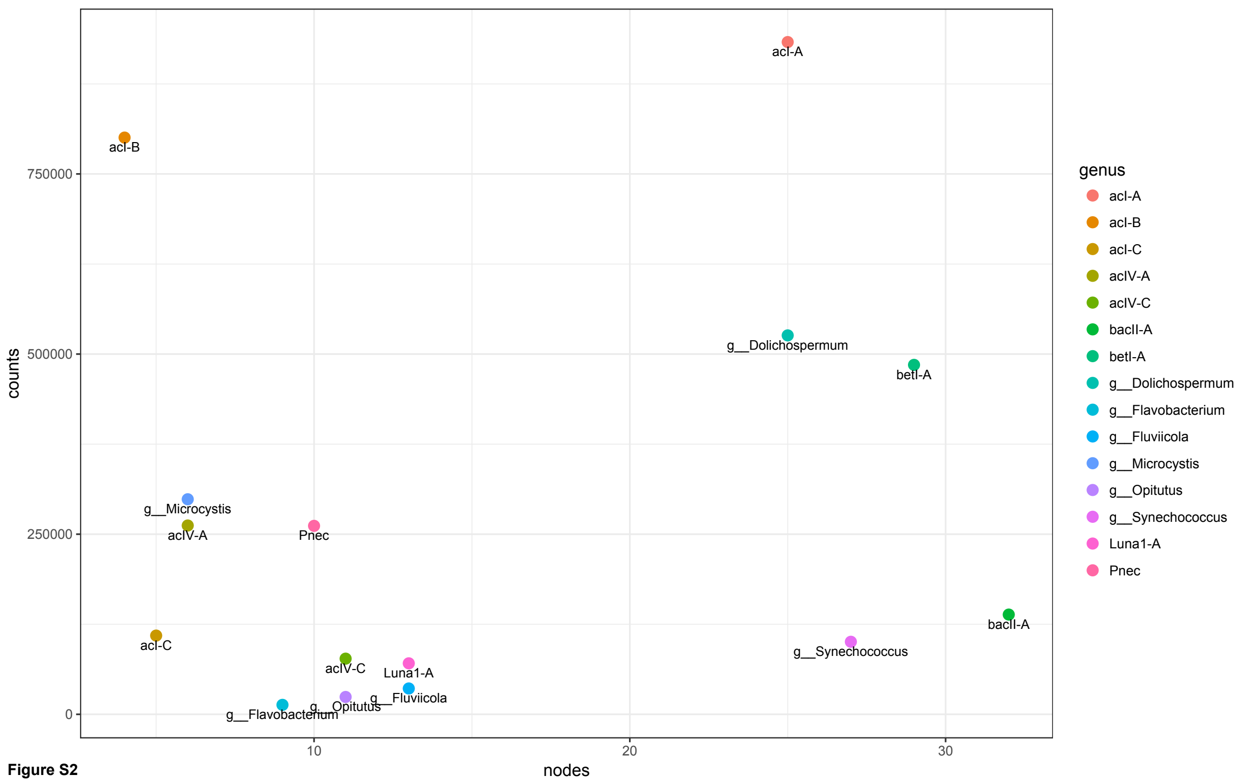

Figure S2. Scatterplot plot illustrating the lack of significant correlation between counts (number of sequence reads per genus) and the number of MED nodes per genus.

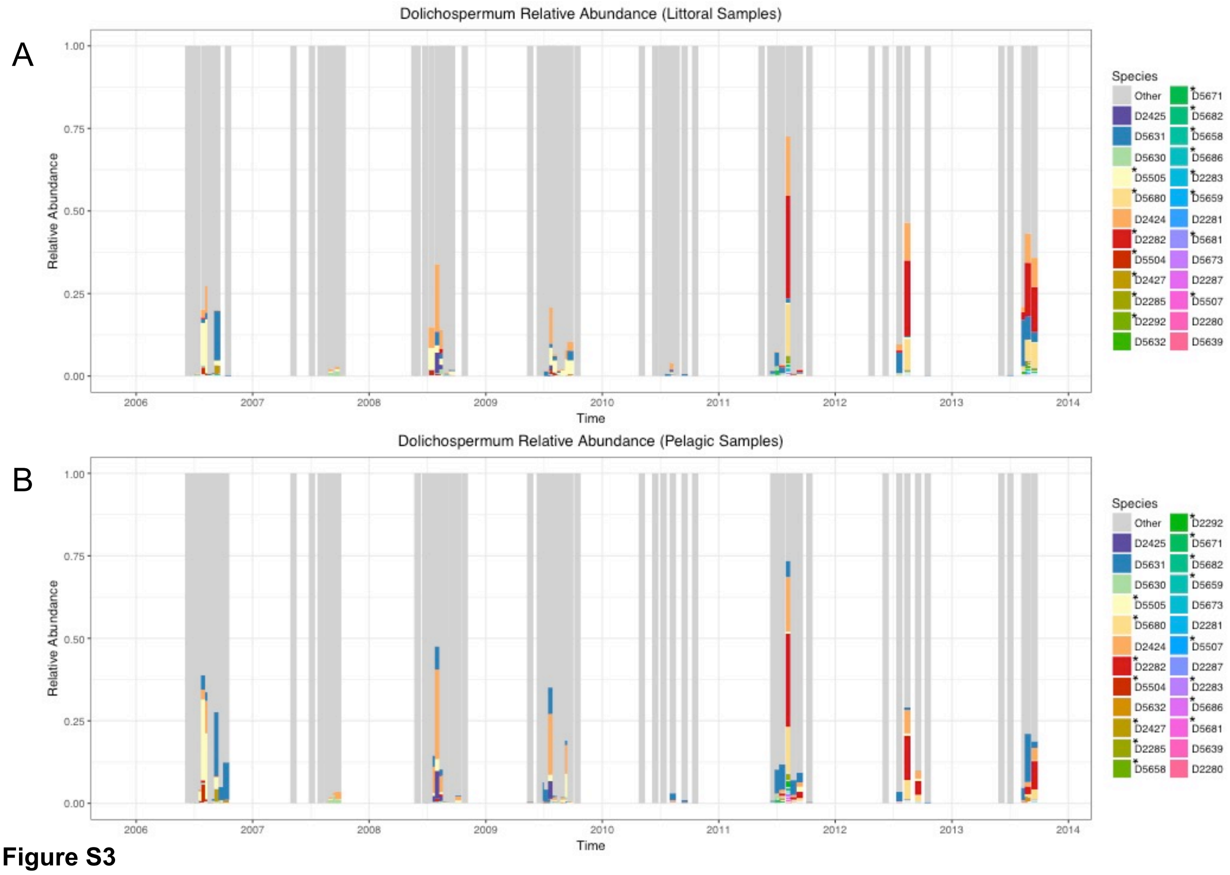

Figure S3

Figure S3. Relative abundance of the different *Dolichospermum* nodes (species) over time.

Conditionally rare nodes are highlighted with an asterisk (*).

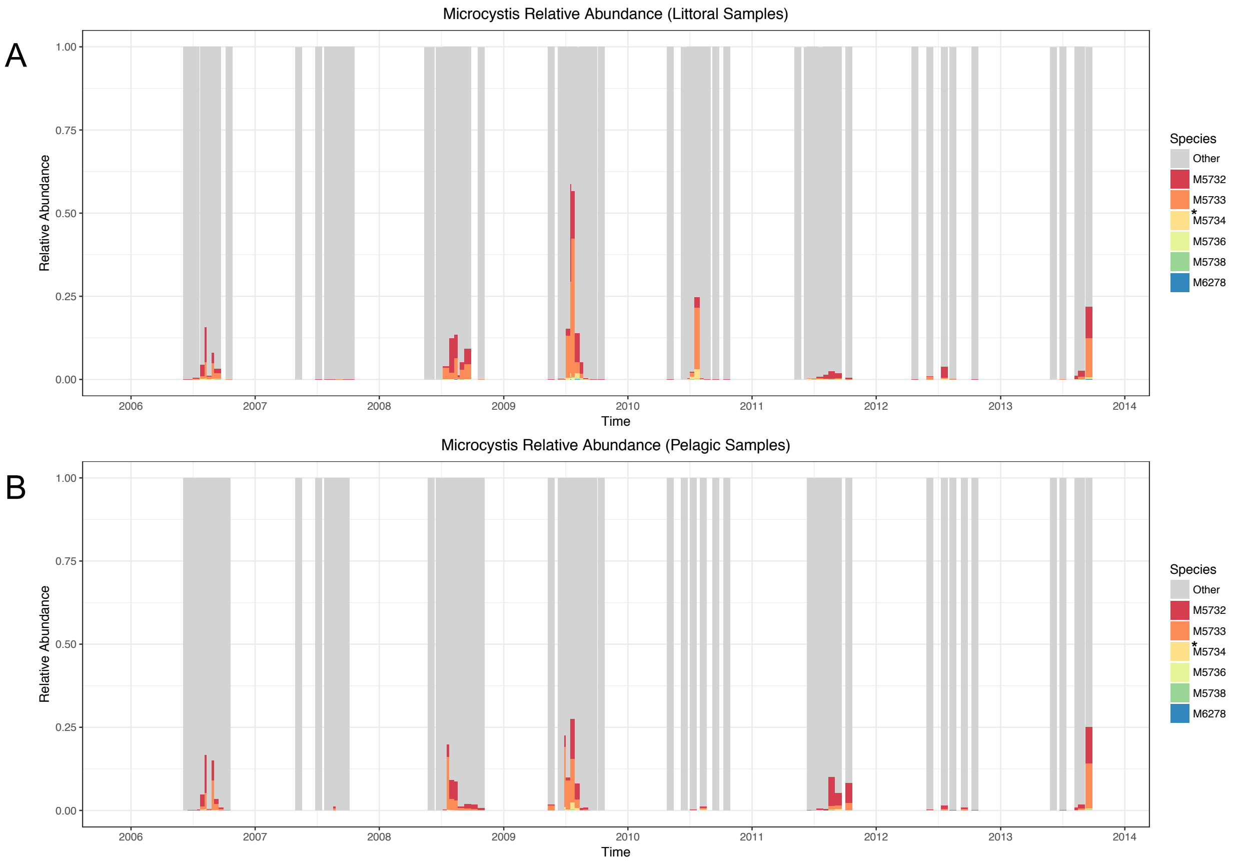

Figure S4

Figure S4. Relative abundance of the different *Microcystis* nodes (species) over time. Conditionally rare nodes are highlighted with an asterisk (*).

Figure S5

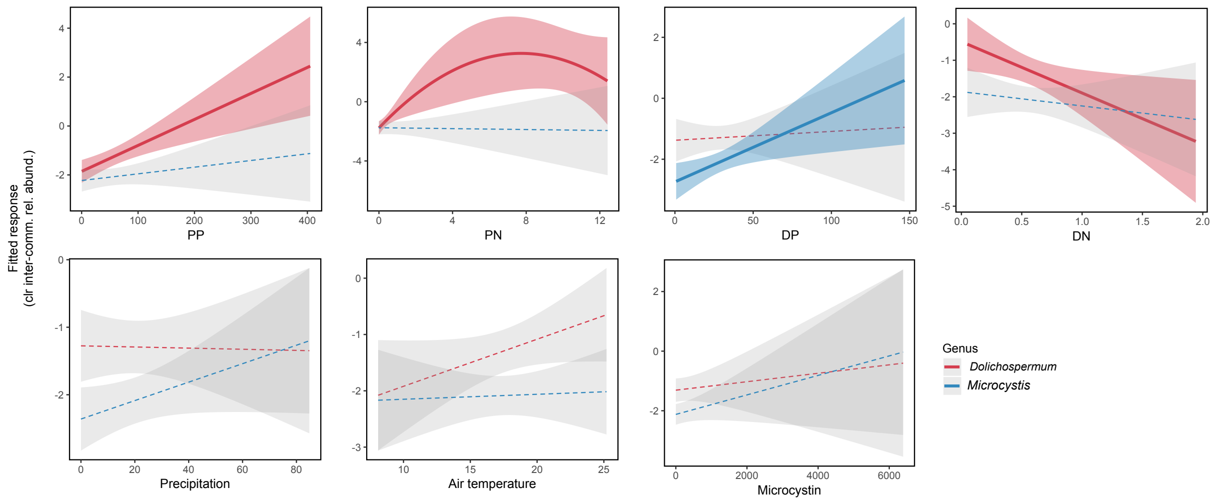

Figure S5. Best-fit polynomial models of the response of *Dolichospermum* (A) and *Microcystis* (B) genera to abiotic factors where the centered-log relative abundance of each genera is expressed in proportion to the total abundance of the bacterial community (clr-inter). Significant relationships are shown by solid lines and coloured confidence intervals. In most cases, the degree-1 polynomial (linear model) provided the best-fit.

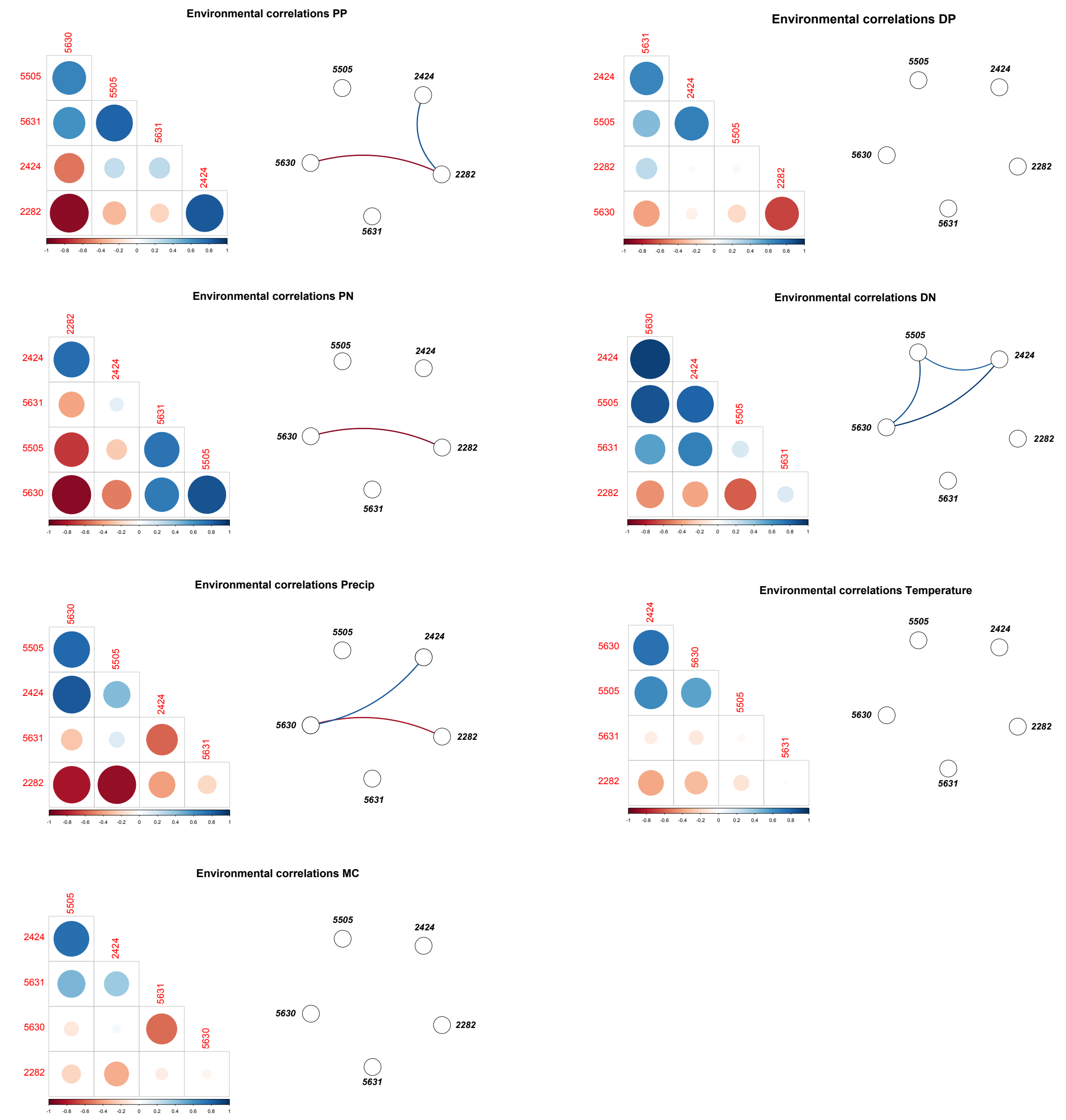

Figure S6

Figure S6. Summary of LVM co-response analysis of *Dolichospermum* nodes to environmental variables. The corrplot (left) shows all correlations among fitted responses, whereas the networks (right) show only the significant correlations among these. The colour of the network lines match those in the corrplot (blues = positive co-response, red = niche separation)

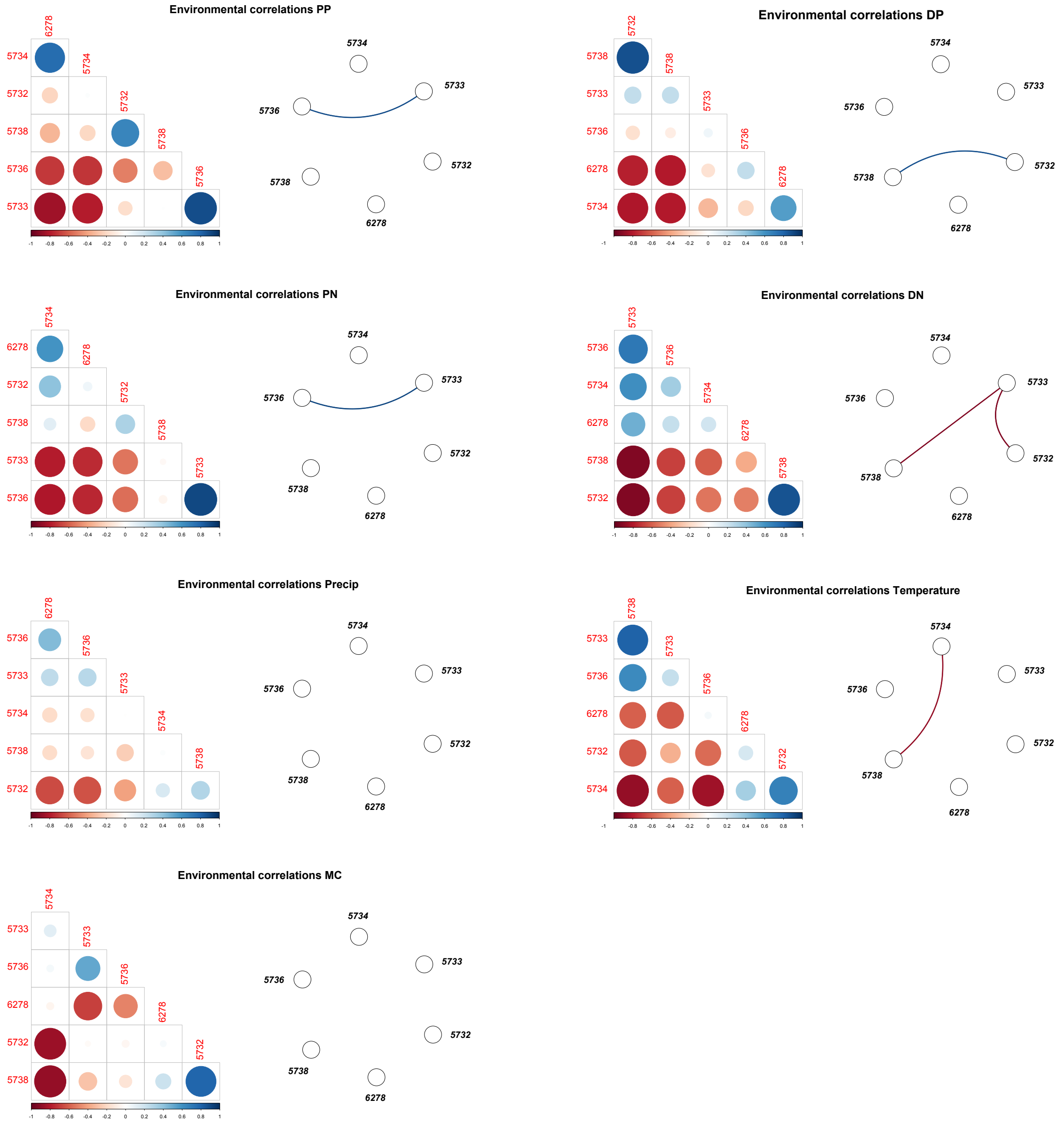

Figure S7

Figure S7. Co-responses analysis of *Microcystis* nodes to environmental variables. See Figure S6 legend for additional details.

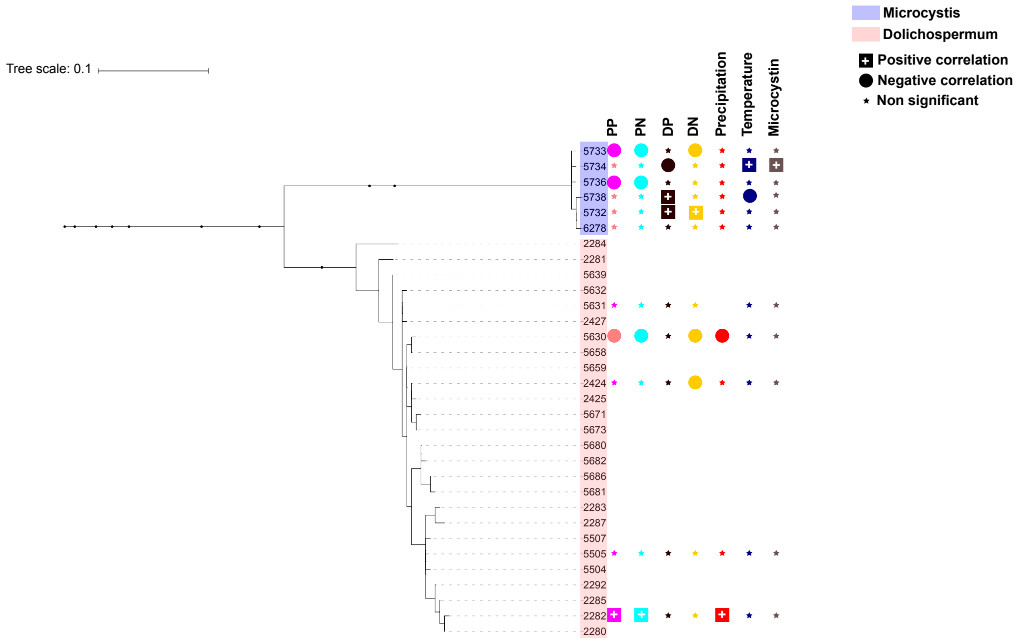

Figure S8. Maximum Likelihood phylogenetic tree of *Dolichospermum* (red) and *Microcystis* (blue) showing the relationship of each *D* and *M* node with the different environmental conditions. Phylogenetic tree was performed using MEGA7 (version 7.0.26).

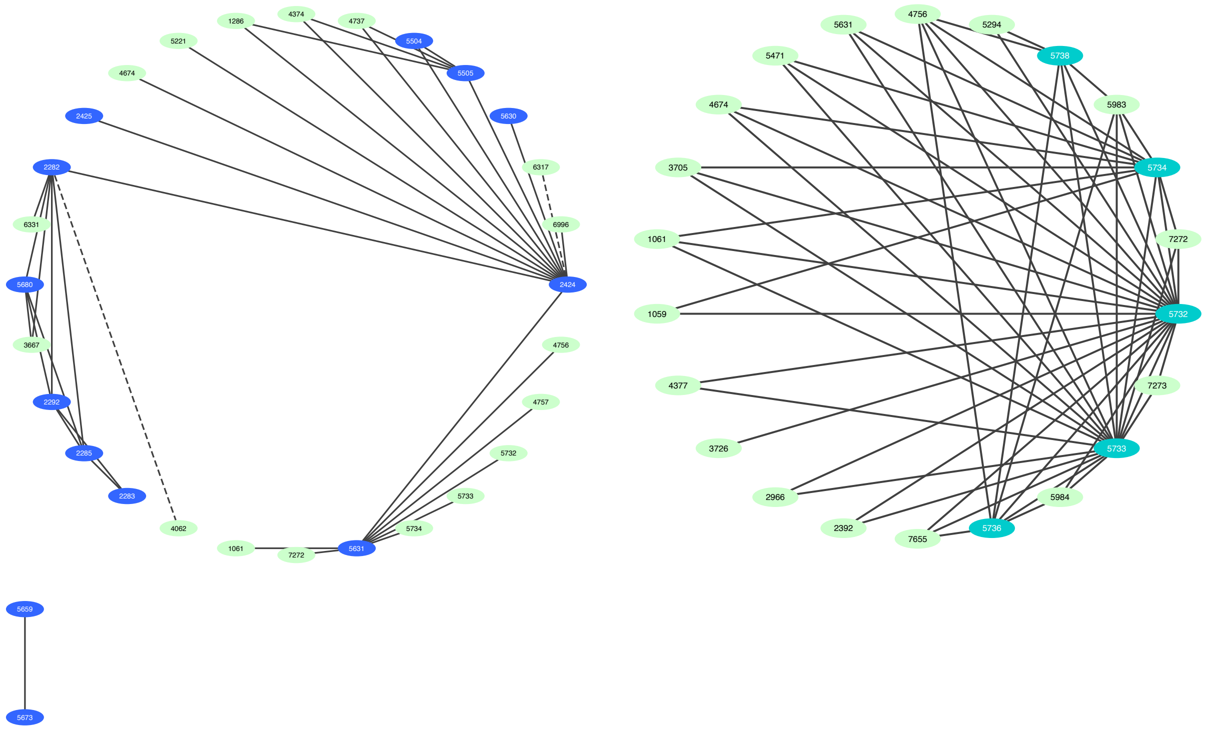

Figure S9. *Dolichospermum* (left) and *Microcystis* (right) SparCC co-occurrence network. Respectively 13/25 *Dolichospermum* and 5/6 *Microcystis* nodes have significant correlations with other taxa. Light green circles indicate taxa that are interacting with *Dolichospermum* or *Microcystis*, blue circles indicate *Dolichospermum* taxa (left), and turquoise circles indicate *Microcystis* taxa (right). Dashed lines show negative correlations.

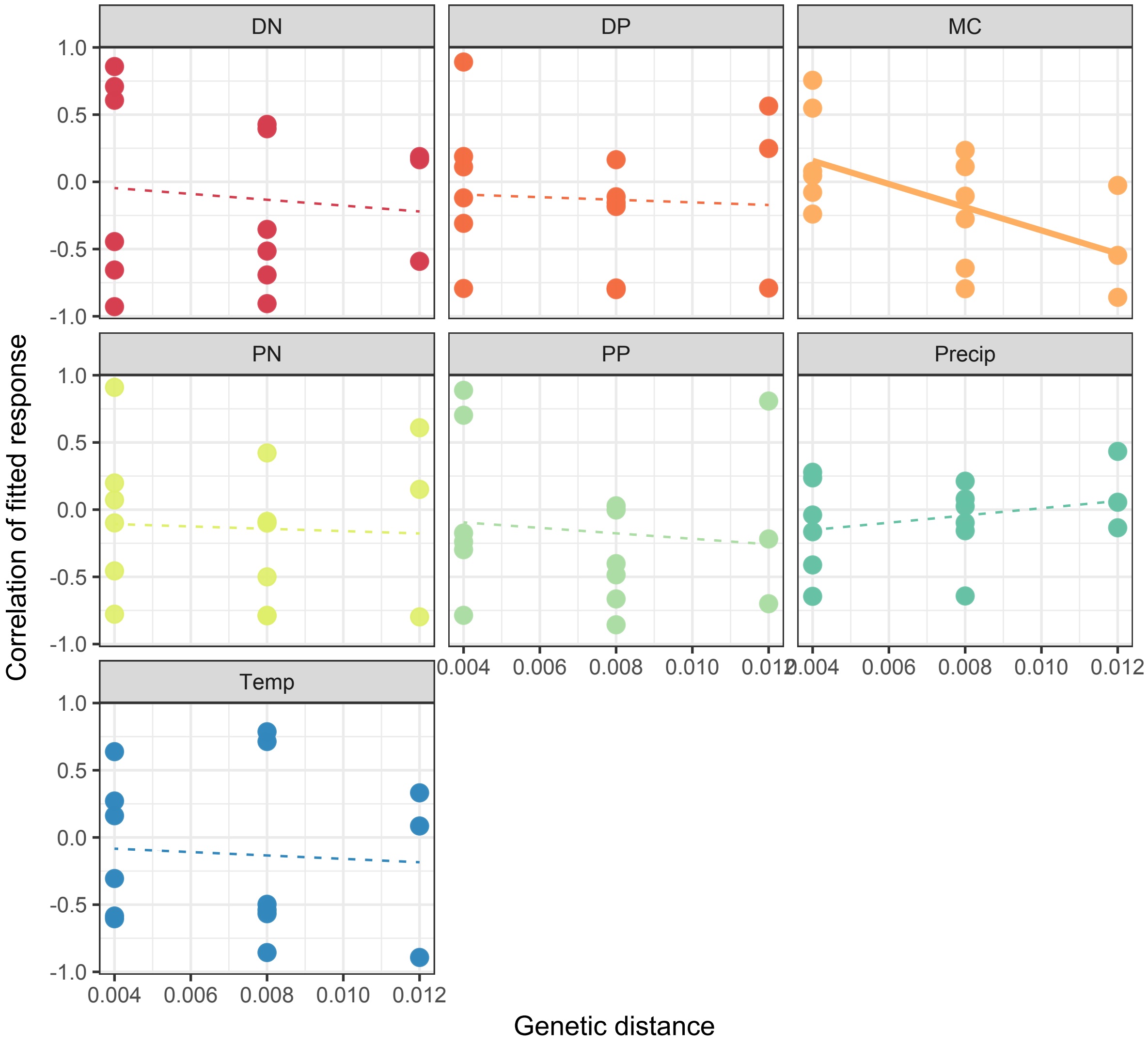

Figure S10. Correlations among the LVM fitted responses of *Microcystis* nodes to each abiotic factor versus their respective pairwise evolutionary distance, where the centered-log relative abundance of each *Microcystis* node is expressed in proportion to the total *Microcystis* abundance (clr-intra). Separate linear and nonlinear models were tested for each abiotic driver; see Table S4 for model results.

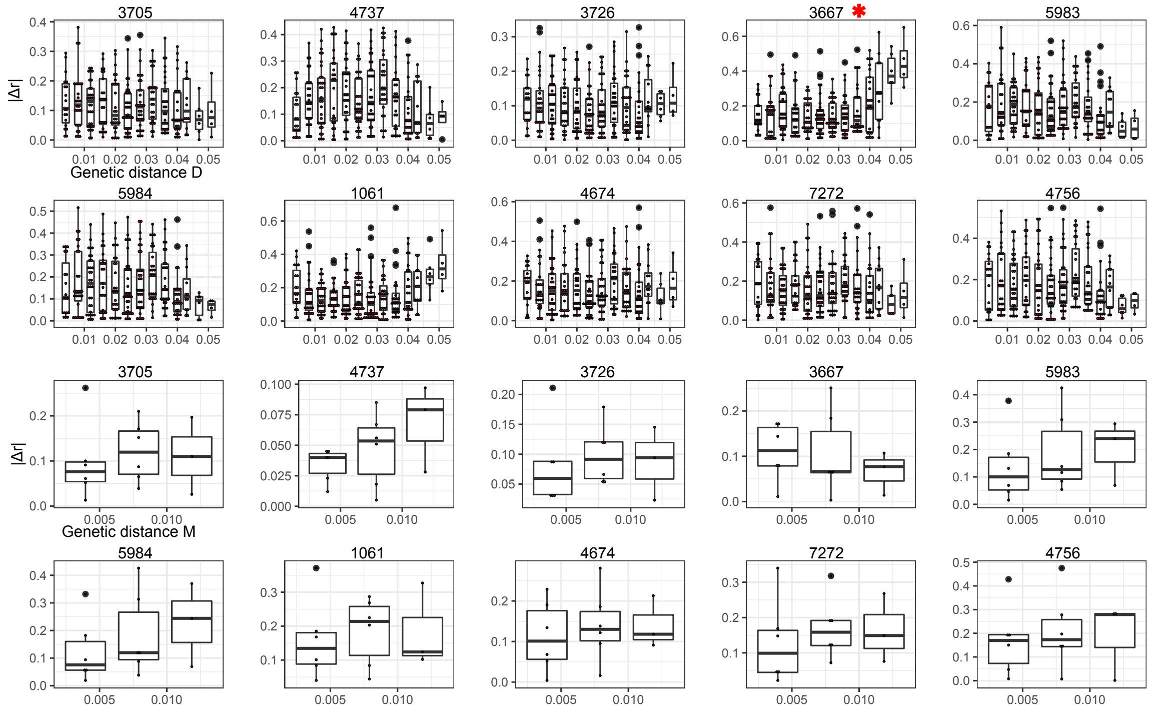

Figure S11. Relationship of co-occurrence between *D* or *M* nodes and different taxa with *D*/*M* genetic distance. The y-axis represents the absolute difference of Sparcc correlation (r) between a *D* or *M* node and a specific taxon T, such that $|\Delta r|$ = | Corr(X1, T) - Corr(X2, T) | where Corr is defined here as the Sparcc correlation score of X (*D* or *M* node) and T (*e.g Cytophagaceae*). The x-axis represents the pairwise genetic distance between *D* or *M* nodes. The red star indicates the only significant relationship (Adjusted R-squared: 0.059, $P < 0.001$).
